# The Photocatalytic Performance of Nd_2_O_3_ Doped CuO Nanoparticles with Enhanced Methylene Blue Degradation: Synthesis, Characterization and Comparative Study

**DOI:** 10.3390/nano12071060

**Published:** 2022-03-24

**Authors:** Fatma El-Sayed, Mai S. A. Hussien, Mervat I. Mohammed, Vanga Ganesh, Thekrayat H. AlAbdulaal, Heba Y. Zahran, Ibrahim S. Yahia, Hosam H. Hegazy, Mohamed Sh. Abdel-wahab, Mohd. Shkir, Santiyagu Valarasu, Medhat A. Ibrahim

**Affiliations:** 1 Nanoscience Laboratory for Environmental and Bio-Medical Applications (NLEBA), Metallurgical Lab.1, Department of Physics, Faculty of Education, Ain Shams University, Roxy, Cairo 11757, Egypt; fatmaelsahaimy@gmail.com (F.E.-S.); a.mohamad9123@gmail.com (M.S.A.H.); vangaganesh@yahoo.com (M.I.M.); 2 Department of Chemistry, Faculty of Education, Ain Shams University, Roxy, Cairo 11757, Egypt; 3 Laboratory of Nano-Smart Materials for Science and Technology (LNSMST), Department of Physics, Faculty of Science, King Khalid University, P.O. Box 9004, Abha 61413, Saudi Arabia; talabdulaal@kku.edu.sa (T.H.A.); dr_hyzahran@kku.edu.sa (H.Y.Z.); dr_isyahia@kku.edu.sa (I.S.Y.); hosam_h_hegazy@yahoo.com (H.H.H.); shkirphysics@gmail.com (M.S.); 4 Research Center for Advanced Materials Science (RCAMS), King Khalid University, P.O. Box 9004, Abha 61413, Saudi Arabia; 5 Semiconductor Laboratory, Department of Physics, Faculty of Education, Ain Shams University, Roxy, Cairo 11757, Egypt; 6 Department of Physics, Faculty of Science, Al-Azhar University, Assiut 71524, Egypt; 7 Materials Science and Nanotechnology Department, Faculty of Postgraduate Studies for Advanced Sciences, Beni–Suef University, Beni–Suef 62511, Egypt; mshaabancnt@gmail.com; 8 PG and Research Department of Physics, Arul Anandar College, Madurai 625514, India; valanroyal@gmail.com; 9 Nanotechnology Research Centre (NTRC), The British University in Egypt (BUE), Suez Desert Road, El-Sherouk City, Cairo 11837, Egypt; medahmed6@yahoo.com; 10 Molecular Spectroscopy and Modeling Unit, Spectroscopy Department, National Research Centre, 33 El-Bohouth Street, Giza 12622, Egypt

**Keywords:** Nd_2_O_3_ doped CuO nanoparticle, nanostructured photocatalyst, methylene blue, visible light, XRD/SEM, photo-Fenton

## Abstract

The growth of the textile industry results in a massive accumulation of dyes on water. This enormous rise in pigments is the primary source of water pollution, affecting the aquatic lives and our ecosystem balance. This study aims to notify the fabrication of neodymium incorporated copper oxide (Nd_2_O_3_ doped CuO) nanoparticles by combustion method for effective degradation of dye, methylene blue (MB). X-ray diffraction (XRD), Field emission Scanning electron microscopy (FESEM), Zeta potential have been applied for characterization. Photocatalyst validity has been evaluated for methylene blue degradation (MB). Test conditions such as time of contact, H_2_O_2_, pH, and photo-Fenton have been modified to identify optimal degradation conditions. Noticeably, 7.5% Nd_2_O_3_ doped CuO nanoparticle demonstrated the highest photocatalytic efficiency, up to 90.8% in 80 min, with a 0.0227 min^−1^ degradation rate. However, the photocatalytic efficiency at pH 10 becomes 99% with a rate constant of 0.082 min^−1^. Cyclic experiments showed the Nd_2_O_3_ doped CuO nanoparticle’s stability over repeated use. Scavenge hydroxyl radical species responsible for degradation using 7.5% Nd_2_O_3_ doped CuO nanoparticles have been investigated under visible irradiation.

## 1. Introduction

**Scheme 1:**
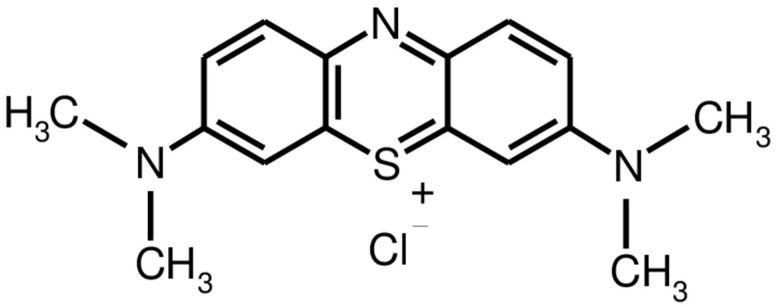
Chemical structure of MB dye.

Over the last decades, because of rapid industrialization, much toxic waste has been released into water bodies. Dyes are involved in various industries, including textile, plastic, skincare products, food processing, medicines, etc. [1]. The dye can negatively impact the aquatic environment due to reduced solar permeation. Furthermore, certain dyes may cause severe illnesses such as mutation and cancer [2]. Significant consequences are the lack of potable water, domestic use, agriculture, farming, etc. [3]. Methylene blue (MB) is a cationic thiazine dye, which forms a blue water solution (see Scheme 1). It is used for various applications, including colored paper, the temporary coloring of cotton, wool, and paper stock dyeing. MB leads to different toxic effects, including eye burning, skin irritation, and gastrointestinal tract. So, it is essential to remediate the dyestuffs wastewater to save water and the environment [4].

Consequently, water pollution must be eliminated [5]. Physical techniques such as adsorption and chemical techniques are employed to remove the textile dyes [6]. The advanced oxidation process includes heterogeneous photocatalysis, low-cost and sustainable technology for water purification that takes place on photocatalyst particles in the presence of photons, causing them to be mineralized [7].

Reusable photocatalysts are used to degrade organic contaminants in photocatalysis. As particle size is reduced, the photocatalyst’s surface area increases. As a result, the number of activating sites has increased, resulting in a higher reactivity. After the absorption of light, the electron has been excited, generating a hole, $O_{2}^{-}$ and ${\mathrm{OH}}^{.}\;$ has been formed due to the reaction between e^−^ with O_2_ and h^+^ with OH^−^ or H_2_O [8]. Many researchers have examined the degradation of various reactive dyes with visible light-assisted Fenton (H_2_O_2_/Fe^2+^ or Fe^3+^) and photo-Fenton (UV-Vis/H_2_O_2_/Fe^2+^ or Fe^3+^) reactions [4]. Both the mechanism and kinetics of the response have been widely explored [9]. Fenton’s reagent, the mixture of H_2_O_2_ and ferrous iron, produces hydroxyl radicals with the ferrous (Fe^2+^) ions, which initiate and catalyze H_2_O_2_ decomposition.

Metal oxide nanoparticles (NPs) such as ZnO, CoO, TiO_2_, SnO_2_, and CuO represent one strategy to lower aqueous organic dyes. In particular, CuO NPs have significant potential because of their low cost, low toxicity, easy availability, optical, catalytic, and antimicrobial characteristics. In addition, copper oxide nanoparticles are noteworthy because of their surface area and increased adsorbed oxygen capacity, leading its bandgap to be ranged from 1.35 to 3.5 eV [10,11]. The strong ability of CuO-NPs for molecular oxygen absorption has a significant impact on the electron-hole pair rearrangement and the removal of the photogenerated electron [12]. Nd_2_O_3_ is rare earth oxide that improves the efficiency of CuO photocatalysis for MB photodegradation in the presence of light with a lower cost [13]. The preparation and characterization of Nd_2_O_3_ NPs have drawn much attention to be studied because of their nano-size structure and surface.

In this work, we synthesized CuO and Nd_2_O_3_ doped CuO photocatalysts with different concentrations of neodymium oxide (Nd_2_O_3_) and characterized by various experimental techniques such as XRD, FESEM, EDX, and DLS. Methylene blue was degraded to examine the photocatalytic activity of the samples. Experiments were performed to enhance the conditions, such as the percentage of Nd_2_O_3_ incorporating CuO, H_2_O_2_, pH, and Photo-Fenton.

## 2. Experimental Techniques

### 2.1. Synthesis of Nd_2_O_3_ Doped CuO Nanostructured

**Scheme 2:**
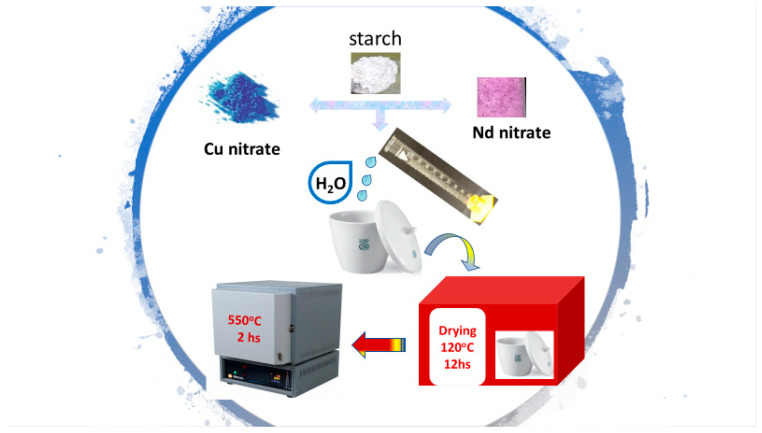
Schematic diagram of the synthesis of Nd_2_O_3_ doped CuO nanocomposites.

The Nd_2_O_3_ doped CuO nanoparticles were prepared by the combustion method. First, 5 g of Copper nitrate (Sigma-Aldrich company, Burlington, MA, USA) has been taken, neodymium nitrate(Sigma-Aldrich company, Burlington, MA, USA) with different weights (0.11, 0.28, 0.58, 0.89, 1.21 g), for (1, 2.5, 5, 7.5, 10%) then 10 g starch and 30 mL distilled water in 6 crucibles and mixed well. Then the crucibles were dried in an oven at 120 °C for 12 h and calcined at 550 °C for 2 h to obtain the proper catalyst, as illustrated in Scheme 2.

### 2.2. Characterization Techniques and Devices

The structure of The Nd_2_O_3_ doped CuO NPs was obtained by a PAN analytical X’Pert PRO, (Philips, Eindhoven, Netherlands) Philips X-ray diffractometer with monochromatic *CuK_α_* operating radiation at 40 kV and 40 mA.

Field emission scanning electron microscopy (FESEM) of The Nd_2_O_3_ doped CuO nanoparticles have been examined by HR-SEM, QUANTA FEG 250, USA (FEI, Hillsboro, OR, USA), to identify their surface morphology.

A Nano Series, Zeta sizer(Malvern Panalytical comapny, MALVERN, UK), has been used to determine the surface potentials. The samples have been suspended at 25 °C into the water to determine the average zeta potential (ζ_av_, mV). In addition, dynamic light scattering (DLS) (Malvern Panalytical comapny, MALVERN, UK), has been used to measure conductivity. The XRD, FE-SEM, and DLS devices are located in the Egyptian Petroleum Research Institute, Naser City, Egypt.

### 2.3. Photocatalytic Performance of Nd_2_O_3_ Doped CuO Nanopowders

**Figure 1:**
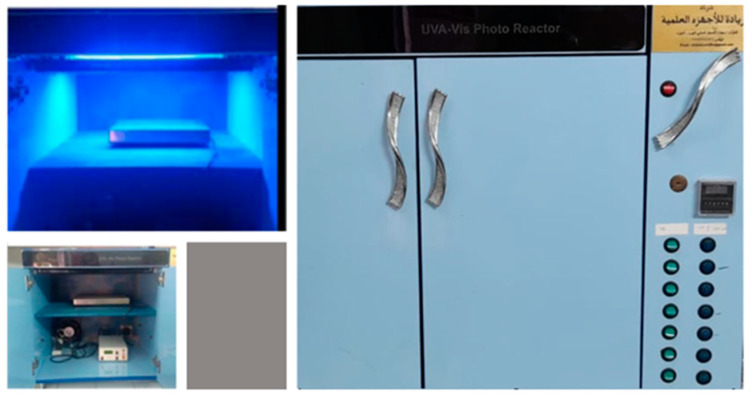
Photoreactor design and operation in NLEBA, Ain Shams University, Cairo, Egypt.

The photocatalytic validity of all prepared samples has been assessed through a wood photoreactor. The photoreactor at NLEBA, Ain Shams University (ASU), was developed by I.S. Yahia and his team [14] (see Figure 1). It stands 100 cm tall and 65 cm broad from the outside. Each of the white and blue bulbs has an output of 18 watts. A 15-point, 500 rpm multi-position magnetic stirrer was used to stir the mixture of Nd_2_O_3_ doped CuO nanopowders and MB solution (Sigma-Aldrich company) at room temperature, allowing us to evaluate many samples at once.

A 100 mg of Nd_2_O_3_ doped CuO NPS was inserted in 50 mL of MB solution. Agitation in darkness results in adsorption-desorption equilibrium. The last step included turning on the white light to shine the prepared samples. The deteriorated solution was taken and centrifuged at ten-minute intervals to remove any nanopowders. To finish, the (LISCO-GmbH) UV–Visible spectrophotometer was used to evaluate the deteriorated solution alongside MB’s stock solution. All operating parameters stay stable during photocatalytic processing.

The degradation of MB dye using white light assisted photo-Fenton reaction was investigated in the presence and absence of 7.5% Nd_2_O_3_ doped CuO catalyst. The photo-Fenton degradation rate was 0.00813 min^−1^ at 0.1 g of Fe^+2^-ions in the presence of 7.5% Nd_2_O_3_ doped CuO, while the rate was 0.00276 min^−1^ at 0.05 g of Fe^+2^-ions in the absence of 7.5% Nd_2_O_3_ doped CuO. This result means that the presence of catalyst enhanced the photo-Fenton degradation rate.

## 3. Results and Discussion

### 3.1. XRD of the Prepared Nanostructured Nd_2_O_3_ Doped CuO

**Figure 2:**
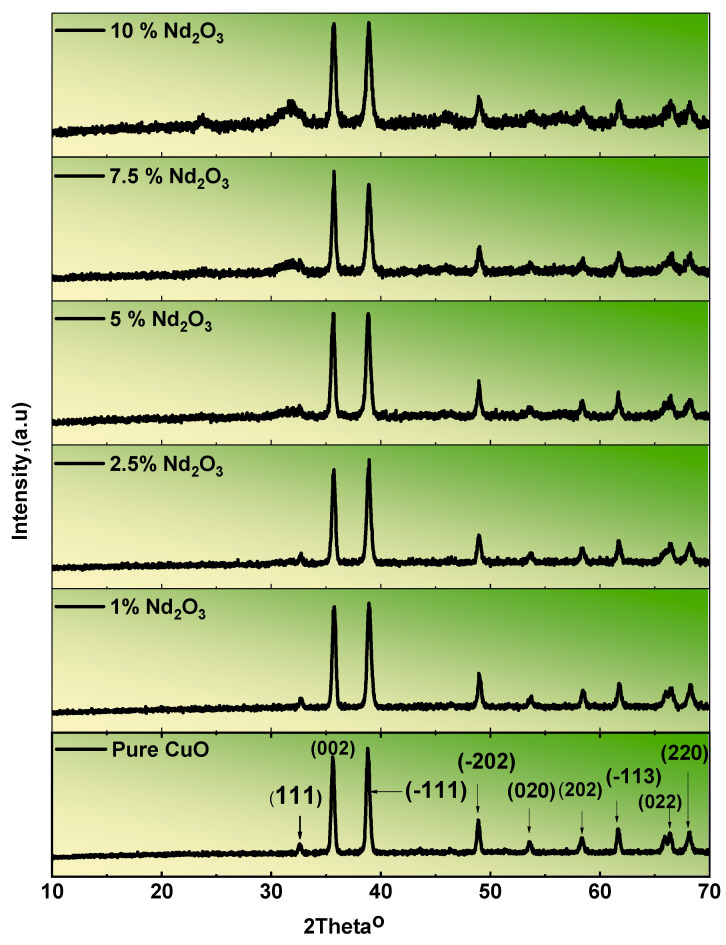
X-ray diffraction patterns for pure CuO and Nd_2_O_3_ doped CuO nanostructured samples.

XRD is the most successful approach for recognizing structural information of Nd_2_O_3_ doped CuO nanoparticles, and it investigated the crystallinity of nanomaterials. The XRD patterns for pure CuO and Nd_2_O_3_ doped CuO nanostructured are shown in Figure 2. The diffraction peaks of pure CuO have been indexed through the characteristic 32.67° (111), 35.63° (002), 38.90° (−111), 48.83° (−202), 53.60° (020), 58.24° (202), 61.59° (−113), 66.5° (022) and 68.19° (220) reflections of the monoclinic phase of CuO [15,16]. The values of diffraction peaks of the as-prepared NPs were tabulated in Table 1.

The intensities and positions of the diffraction peaks for the CuO NPs agree with the reported data (JCPDS 048-1548). Indexes the peaks of the studied samples, and no peaks of impurities are found in the XRD pattern. It could be observed that the Nd_2_O_3_ doped CuO NPs had the same XRD design as the CuO, indicating the crystalline structure of the photocatalyst. The excellent dispersion of Nd_2_O_3_ via CuO, which acts as the host crystal, has also been confirmed. A small secondary phase appeared due to incorporating Nd_2_O_3_ with different percentages (1, 2.5, 5, 7.5, and 10%) into the CuO matrix. Two crystalline peaks appeared at 2-theta: 23°, and 32° with *hkl* = [110] and [011]. They correspond to the peaks of Nd_2_O_3_ as hexagonal phase (JCPDS card No. 01-074-2139). These two peaks strongly appeared at 10% Nd_2_O_3_ doped CuO nanoparticles [13,17]. The equation of Scherrer was used to compute crystallite size [18].

(1) $$D=\frac{0.9\lambda }{\beta \;\cos\theta },$$

In this case, D represents the crystallite size and X-ray wavelength (λ); FWHM represents the full width at half maximum (FWHM). The lattice strain (ε) was determined using the following method [18]:

(2) $$\varepsilon =\frac{\beta \cos\theta }{4},$$

Dislocation density (δ) has been achieved by [18,19]:

(3) $$\delta =\frac{1}{D^{2}}$$

Moreover, where the diffraction peak angle is θ. The calculated mean values of the grain size (D) are tabulated in Table 2. The first phase’s average crystallite domain size ranges from 22.0 to 30.1 nm, while the second phase ranges from 27.1 to 36.7 nm.

The value of (D) for CuO is found in the range of 20–30 nm. It was found that (D) of Nd_2_O_3_ sample with Scherrer formula is about 35 nm. The ε and δ were calculated and tabulated in Table 2. It was found that Nd_2_O_3_ and CuO atoms bond with O^−^ atoms by increasing Nd_2_O_3_ content. Lattice strain (ε) arises from the slight variation in the atomic radii of Nd^2+^- and Cu^2+^- beside Nd_2_O_3_ content, where Cu^2+^ is replaced with Nd^2+^ in the host lattice. The decrease in lattice strain (1.2 × 10^−3^) at 7.5% Nd_2_O_3_ doped CuO nanoparticle (Table 2) because of atomic radii and dopant concentration reduces local distortion of the crystal lattice. The crystallite size (30.1 nm) of the 7.5% of Nd-CuO sample was increased because of a decrease in δ (1.4 × 10^−3^ nm^2^) and intrinsic micro-strain [20].

### 3.2. FESEM Analysis of Nd_2_O_3_ Doped CuO Nanostructured

**Figure 3:**
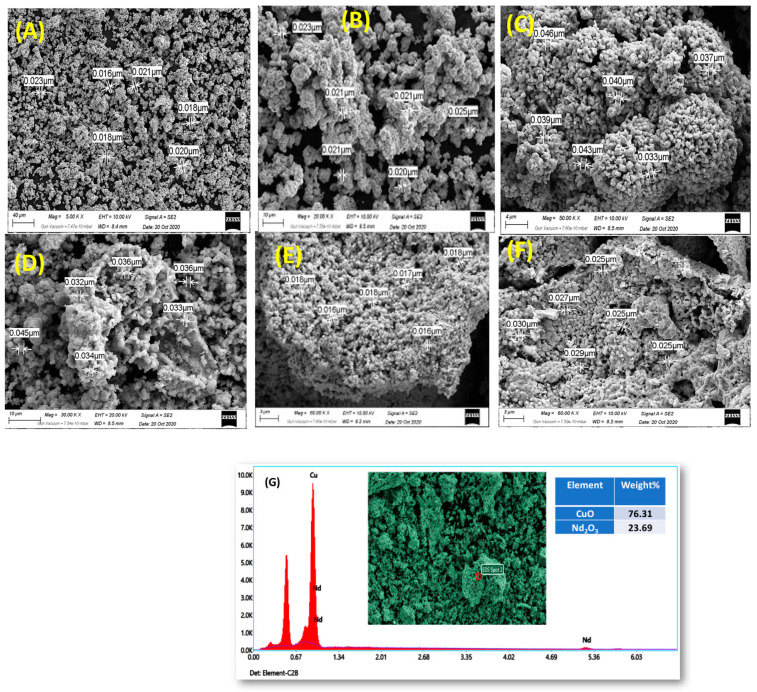
(**A**–**F**): FE-SEM images of pure CuO and Nd_2_O_3_ doped CuO nanostructured samples, (**G**) EDAX spectrum of 7.5% Nd_2_O_3_ doped CuO nanostructured sample.

The efficiency and activity of photocatalysts depend on the morphological parameters [21]. Therefore, the morphology characterization of the CuO nanoparticles was investigated by FESEM, as shown in Figure 3A. The nanoparticles have a uniform size, spherical shape, and appropriate separation [22,23]. Figure 3B–F exhibit the FESEM images of Nd_2_O_3_ doped CuO NPs at various concentration of Nd_2_O_3_. It is evident that the particles had quasi-spherical shapes and aggregated homogeneously. Empty spaces between the particles are pores arising from the inter-aggregation of particles [15]. From the FESEM result, the average crystalline size of the prepared nanocomposite materials was found in the range of 17–39 nm. As shown in the Table 3, 7.5%Nd_2_O_3_ doped CuO has the smallest value of particle size (17 nm), which means that increasing surface area of catalyst thereby, the improvement of photodegradation efficiency of catalyst [15].

EDX image depicts the peaks of CuO and Nd_2_O_3_ beside atomic percentage of elements (see inset of Figure 3G) contained in 7.5% Nd_2_O_3_ doped CuO nanoparticle, where it verified the production of Nd_2_O_3_ doped CuO nanoparticle by homogeneous chemical structure.

### 3.3. DLS of Nanostructured Nd_2_O_3_ Doped CuO

**Figure 4:**
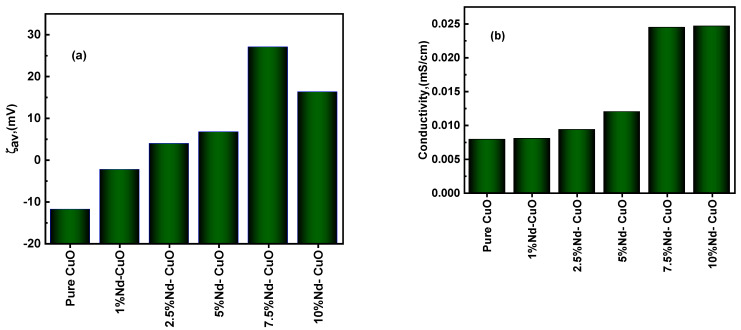
(**a**) Average zeta potential values, (**b**) conductivity of the samples.

Figure 4a,b depicts the average ζ_av_ and conductivity obtained from % Nd_2_O_3_ doped CuO nanoparticles. As shown in Figure 4a, ζ_av_-potential increased by increasing percentage Nd till 7.5% Nd_2_O_3_ doped CuO nanoparticle reaching ~27 mV, which it can attribute to the deformation in the CuO crystal lattice by increasing content of Nd_2_O_3_. Figure 4b depicts the conductivity enhancement by increasing Nd_2_O_3_ content, which was 0.025 mS/cm at 7.5% Nd_2_O_3_ doped CuO nanoparticle. Enhancement of conductivity can be attributed to an increase of ζ_av_-potential value where Photocatalytic activity increases up to 7.5 percent with the increasing mobility of holes causing a photocatalytic activity enhancement [21].

### 3.4. Photocatalysis Using Nanostructured Nd_2_O_3_ Doped CuO

#### 3.4.1. Effect of Nd^+3^ Doping

**Figure 5:**
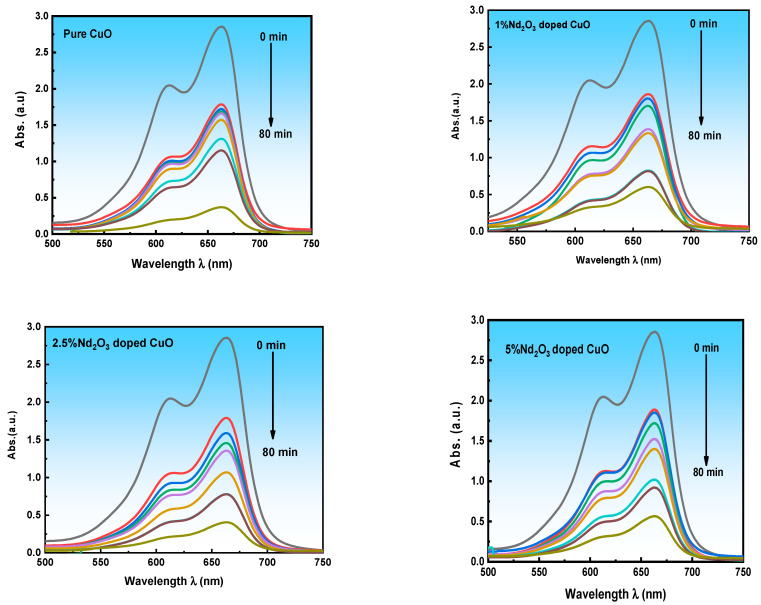

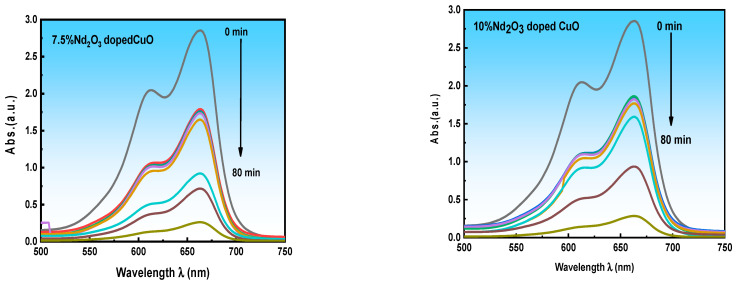
Absorption spectra of MB dye under visible light irradiation with pure CuO and five different concentrations of nanostructured Nd-doping CuO.

**Figure 6:**
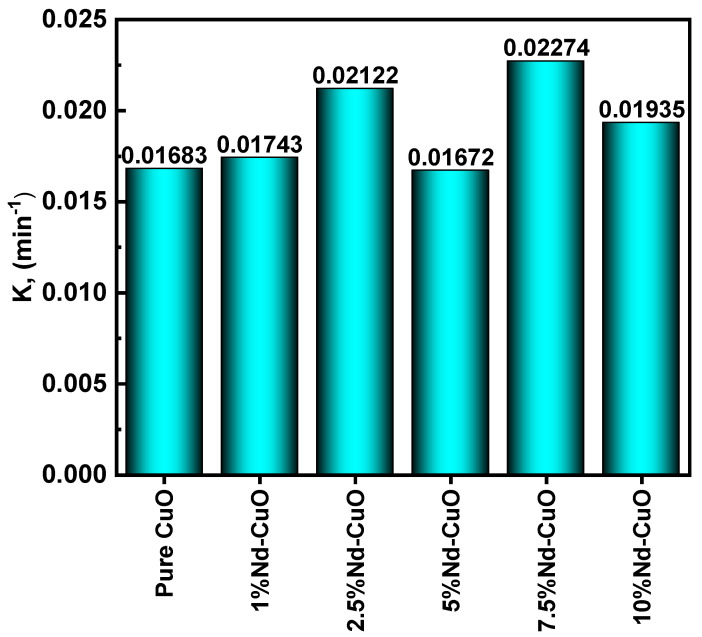
Rate constant values of MB dye photodegradation reaction in the presence of pure CuO and Nd_2_O_3_ doped CuO NPs with different concentrations of Nd_2_O_3_.

The as-prepared nanomaterials (100 mg) have been subjected to visible (80 min irradiation) photodegradation of MB dye. Figure 5 illustrates the MB absorption spectra in the presence of visible illumination with pure CuO and Nd_2_O_3_ doped CuO nanoparticles. The λ_max_ of MB is 664 nm. It is obvious from Figure 5 that the intensity of all samples decreases with increasing time, which means that the dye’s photodegradation rate increased with time. The results revealed that the pure CuO catalyst achieved 87% degradation of MB, while 7.5% Nd_2_O_3_ doped CuO catalyst achieved the highest photocatalytic efficiency, up to 90.8% within 80 min under visible light irradiation. The rate of MB degradation k (min^−1^) was achieved by [24]:

(4) $$\ln\left(\frac{A}{A_{o}}\right)=\mathrm{kt}$$

where absorption at 0 min illumination is A_o_ (mg.L^−1^), and absorption at irradiation time t (min), is A. The k values for CuO and Nd_2_O_3_ doped CuO nanoparticles are depicted in Figure 6. The higher the doping level, the higher the rate of deterioration up to 7.5% Nd_2_O_3_ doped CuO nanoparticle, while at 10% Nd_2_O_3_ doped CuO nanoparticle, the degradation begins to decrease, which means that 7.5% Nd_2_O_3_ doped CuO nanoparticle has the highest degradation rate constant of 0.02274 min^−1^ (as seen in Table 4).

#### 3.4.2. Influence of H_2_O_2_ Concentration of 7.5% Nd_2_O_3_ Doped CuO Nanoparticle

**Figure 7:**
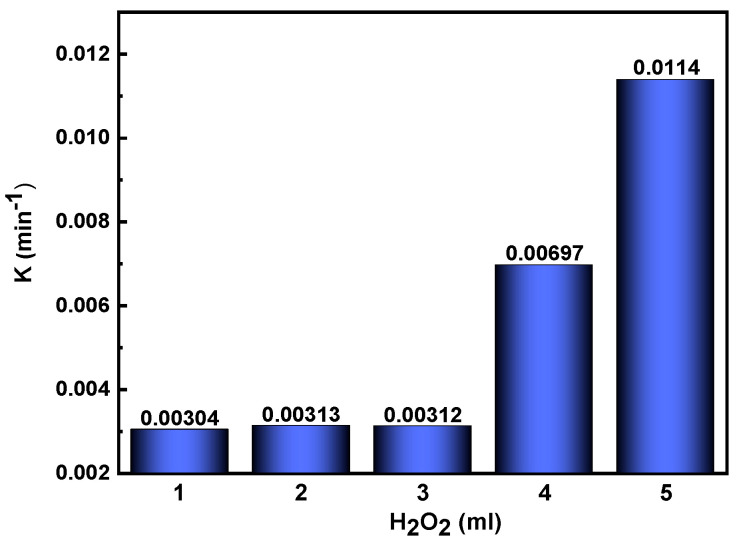
The k versus H_2_O_2_ concentration of MB dye by 0.1 g of 7.5% Nd_2_O_3_ doped CuO NPs under visible irradiation.

The synthesis of HO^−^ is based on H_2_O_2_, one of the cleanest and optimal oxidants for the dye’s degradation and organic pollutants in wastewater [24]. In this investigation, five different concentrations were utilized of H_2_O_2_ (1, 2, 3, 4 and 5 mL) in the presence of 0.1 g of 7.5% Nd_2_O_3_ doped CuO nanoparticle for visible photodegradation of each one of MB dye. Figure 7 depicts the effect of H_2_O_2_ concentration on photodegradation. As can be seen, the photodegradation rate increases with increasing H_2_O_2_ concentration until it reaches its maximum at 5 mL (as shown in Table 4). According to Equation (5), the greater reaction rates following the addition of H_2_O_2_ are due to an increase in the concentration of hydroxyl radical [25]:

(5) $$H_{2}O_{2}+\mathrm{OH}\to {\mathrm{HO}}_{2}+{\;H}_{2}O,$$

As a result, adding hydrogen peroxide at the required quantities can speed up the photodegradation of MB dye.

#### 3.4.3. Effect of pH on 7.5% Nd_2_O_3_ Doped CuO Nanoparticle

**Figure 8:**
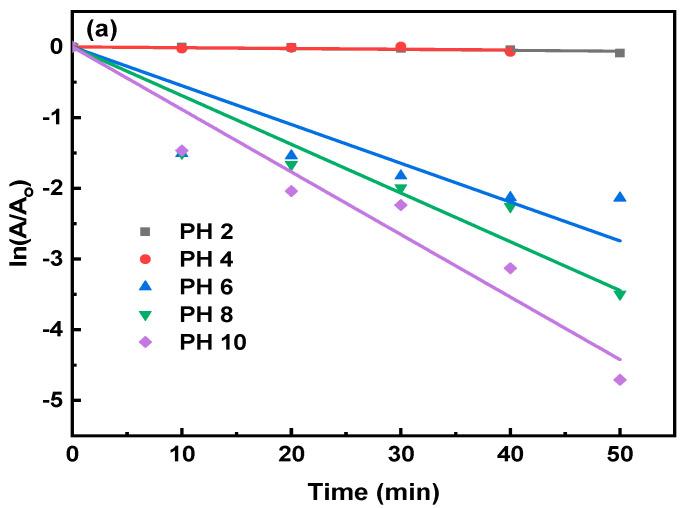

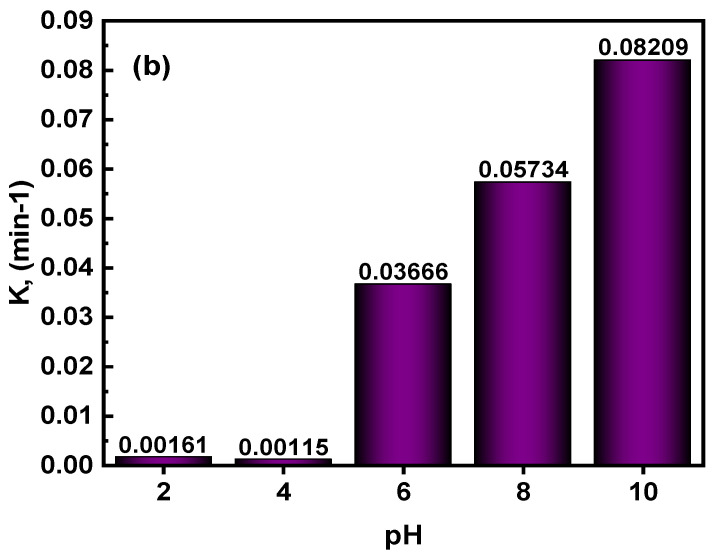
The different pH concentrations using 0.1 g of 7.5% Nd_2_O_3_ doped CuO nanoparticle in the presence of MB dye under visible irradiation, (**a**) Kinetics (**b**) The rate constant.

Due to the high pH dependency of certain aspects, such as semiconductor surface charge state and dissociation of chemicals in the solution, the pH of the solution impacts photodegradation processes. Therefore, photodegradation was studied in ideal circumstances with a pH range of 2 to 10 to evaluate the impact of pH on the photodegradation process. Figure 8a,b represents kinetic study and the rate constant (k) of photodegradation of MB at different pH values in the presence of 7.5% Nd_2_O_3_ doped CuO nanoparticle as a catalyst. 

**Scheme 3:**
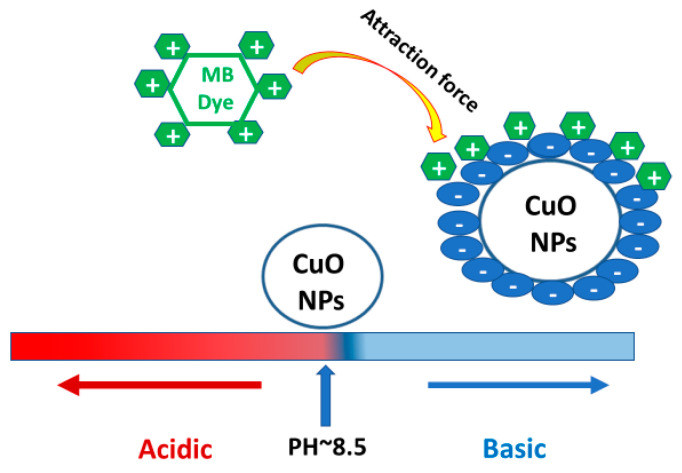
Role of zero-point charge of CuO NPs on the degradation of cationic dye.

The result exhibited a maximum efficiency (99%) at pH 10 after 50 min from the start of irradiation (as seen in Table 4). Several techniques were utilized to calculate the pH of the CuO NPs’ point of zero charges (pH_pzc_), which was between 8.5 and 9.5 [26,27]. The charge on the photocatalyst surface was positive and negative, below and above pH_pzc_, respectively. When the pH is less than pH_pzc_, repulsion between the positively charged 7.5% Nd_2_O_3_ doped CuO catalyst and cationic dye reduces photodegradation efficiency [27]. Scheme 3 depicts the impact of CuO NPs’ pH_pzc_ on cationic dye degradation. Moondeep et al. [26] At greater pH, the CuO NP surface is charged negatively, which improves its interaction with cationic dye (VB). Meanwhile, the CuO NP surface is charged positively at lower pH, enhancing its interaction with anionic dye.

#### 3.4.4. Effect of Fenton and Photo-Fenton on MB Photodegradation

**Figure 9:**
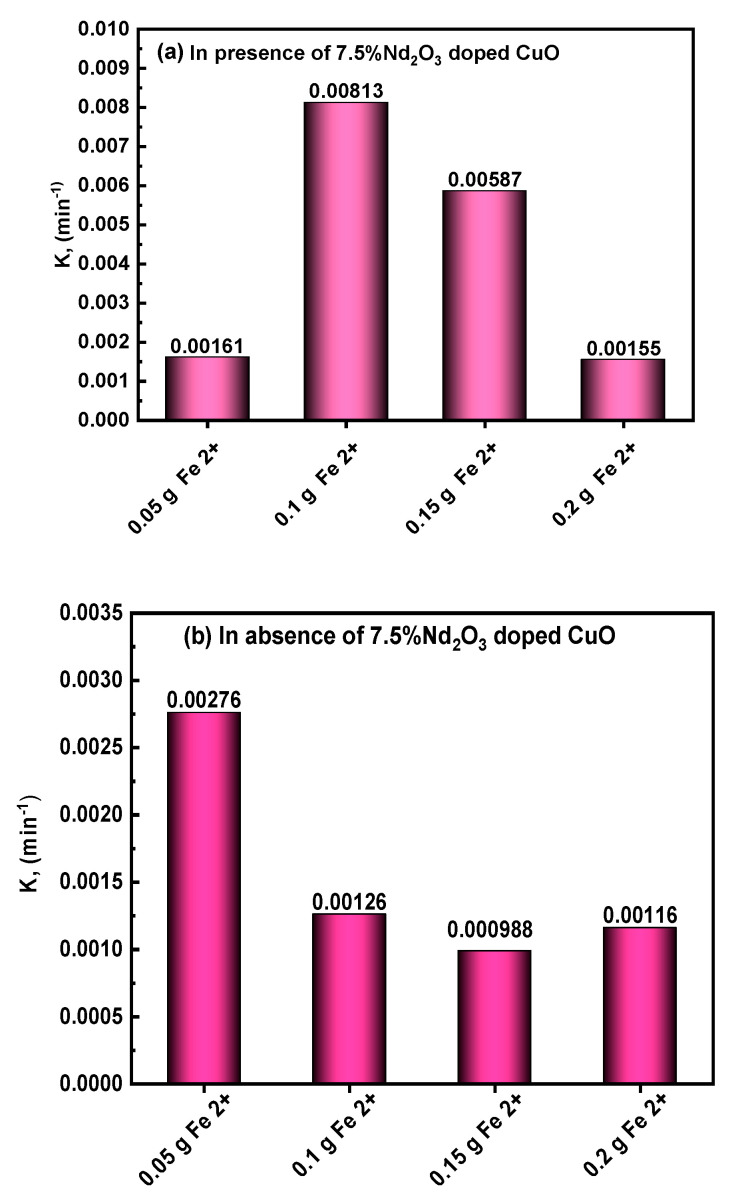
The rate constant Photo-Fenton, (**a**) with and (**b**) without 7.5% Nd_2_O_3_ doped CuO nanocomposites.

Fenton and Photo-Fenton processes have been demonstrated to be the most promising strategies for wastewater treatment. These techniques contribute to improving the environment through a reduction in the pollution resulting from complicated dye molecules and other organic substances as a green chemical and ecological method [9]. The current study investigates photochemical degradation of MB dye by photo-Fenton reaction in the presence and absence of 7.5% Nd_2_O_3_ doped CuO catalyst. Figure 9a shows the effect of ferrous sulfate concentration [0.05 to 0.2 g Fe^+2^-ions] on the rate of photochemical degradation in the presence of 7.5% Nd_2_O_3_ doped CuO catalyst, maintaining all other parameters constant. The findings displayed in Figure 9a enhance the photo-Fenton degradation rate by increasing the content of Fe^+2^-ions up to 0.1 g by a continuous rate of 0.00813 min^−1^. Increasing the number of Fe^+2^-ions up to this specific Fe^+2^ concentration improves ^•^OH radical production and increases the photochemical degradation rate. In addition, the reaction rate was reported to be lowered with increasing Fe^+2^-ions concentration. Figure 9b shows the effect of Ferrous sulfate concentration [0.05 to 0.2 g Fe^+2^-ions] on the rate of photochemical degradation in the absence of 7.5% Nd_2_O_3_ doped CuO catalyst.

The result showed that the optimum rate constant was 0.00276 min^−1^ at 0.05 g of Fe^+2^ ions concentration without a catalyst. The constant rate in the presence of a catalyst is higher than the absence of them. The rate constant (*k*) values and degradation (%) at different Nd_2_O_3_ concentrations, H_2_O_2,_ and pH were calculated and tabulated in Table 4.

The reaction in Fenton was initially identified by H.J. Fenton [28] and is classified as an improved oxidative potential for H_2_O_2_ in acidic media when iron (Fe) is a catalyst. The reactions included in Fenton processes are [29]:

(6) $${\mathrm{Fe}}^{2+}+H_{2}O_{2}\to {\mathrm{Fe}}^{3+}+{\mathrm{OH}}^{.}+{\mathrm{OH}}^{-},$$

(7) $$H_{2}O_{2}\to {\mathrm{HO}}_{2}^{.}+H^{+},$$

(8) $${\mathrm{Fe}}^{2+}+{\mathrm{OH}}^{.}\to {\mathrm{Fe}}^{3+}+{\mathrm{OH}}^{-},$$

(9) $${\mathrm{Fe}}^{3+}+{\mathrm{HO}}_{2}^{.}\to {\mathrm{Fe}}^{2+}+O_{2}+H^{+}$$

(10) $${\mathrm{OH}}^{.}+{\mathrm{OH}}^{.}\to H_{2}O_{2},$$

(11) $$\;\mathrm{MB}\;\mathrm{Dye}+{\mathrm{OH}}^{.}\;\to \mathrm{Degraded}\;\mathrm{Product}$$

#### 3.4.5. Comparison of the Photocatalytic Activity of Several Compounds Based on CuO

In the present work, 7.5% Nd_2_O_3_ doped CuO nanocomposite exhibited the highest photocatalytic efficiency, up to 90.8% in 80 min, with a 0.0227 min^−1^ degradation rate. At pH 4, the degradation efficiency reaches its maximum value to be 99%. When comparing the data acquired with other research, the capacity to study, evaluate and contrast outcomes may be demonstrated. Table 5 indicates the comparison of photocatalytic degradation of MB and other dyes in the presence of Nd-doped metal oxide with different previous work samples. M. Arunpandian et al. [30] found that 0.3 wt% of Nd_2_O_3_-doped ZnO was the highest active, showing high photocatalytic activity for the degradation of MB dye. Arun Pandian M. et al. [16] It was shown that MB degradation was by Ag/Nd_2_O_3_-ZnO Nanocomposite within 30 min with a 98.12 percent efficiency under visible light. Dhanya et al. [31] Including Nd^3+^ in the SnO_2_ lattice decreases bandgap and inhibits the electron-hole recombination. Thus, visible light can excite electrons from the valence band to the conduction band. Samuel et al. [32] Nd–ZnO showed good photocatalytic activity. In comparison to ZnO, nanocomposite [Nd–ZnO–GO (0.3% of Nd)] demonstrated higher photocatalytic efficiency and could be regarded for promise as a photocatalyst in organic wastewater treatment. Samuel Osei-Bonsu Oppong et al. [33] The photodegradation efficiency of indigo carmine solutions exposed to simulated solar light for 3 h with Nd–TiO_2_–GO (0.6 percent Nd) nanocomposites was found to be 92 percent. Umair Alam et al. [34] showed that the Nd-doped ZnO is the best activity with 98% degradation efficiency compared used other rare earth metals. Sonali et al. [35] The optimum photocatalytic activity for MB degradation was ob-served for 3.0 mol. Percent Ce-CuO, which led to 98 percent degradation in 180 min. Xia Shao et al. [36] When the ratio of Nd to TiO_2_ is 3–4 wt%, the highest activity is reached, and MB is degraded by UV- light in 160 min. Reda M. Mohamed et al. [37] For 2 h, the photocatalytic efficiency of mesoporous 3 percent Nd_2_O_3_/ZnO for TC degradation reached 100%. Moondeep et al. [26] under diverse energy sources, such as UV-visible irradiation and UV radiations, the degradation efficiency of CuO NPs on cationic (VB) and anionic (DR) dyes were measured.

#### 3.4.6. Reusability and Stability

**Figure 10:**
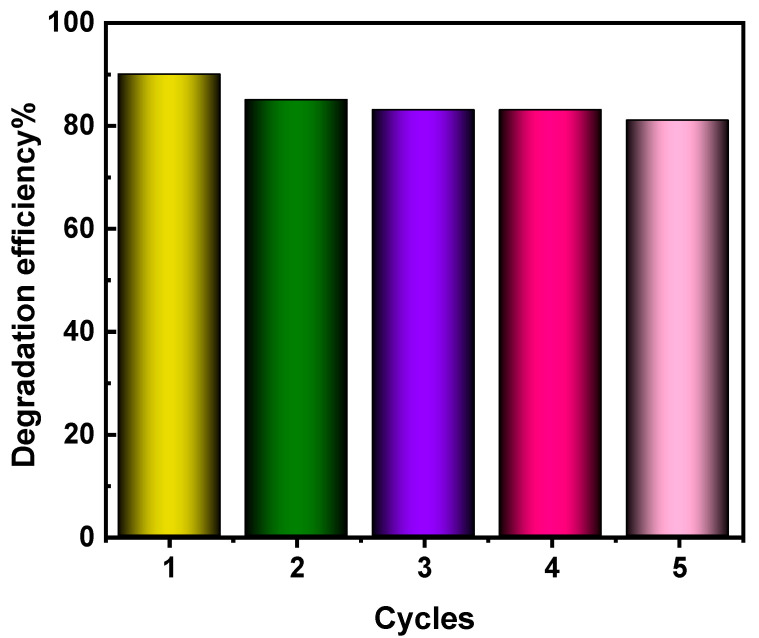
Recycling of 7.5% of nanostructured Nd_2_O_3_ doped CuO.

In practical applications, the photostability of a photocatalyst is an essential feature. The synthesized Nd_2_O_3_ doped CuO photocatalyst was exposed to five photocatalytic trial runs to determine its photostability by adding the reused photocatalyst to new MB solutions under the same experimental conditions. The photocatalyst was reused after centrifugation without regeneration. Figure 10 depicts the recycling of 7.5% Nd_2_O_3_ doped CuO nanostructured. It was found that, in five successive experimental runs, photocatalyst activity reached 88% of MB degradation, which promotes the prepared samples for its photocatalytic performance in environmental treatment.

#### 3.4.7. Influence of Radicals’ Scavengers on Photocatalytic Activity

The photocatalytic reaction occurs in 3 stages: absorption of light, generation and separation of electron-hole, and oxidation-reduction response on the photocatalyst’s surface. e^−^ and h^+^ are extremely recombined so, the trapping and transfer of surface charges are limited. Therefore, it is vital to eliminate the recombination of surface charge and promote charge transfer to the surface-active sites to boost the carriers’ dynamics and photocatalytic activity. There have thus been significant attempts to increase the effectiveness of photocatalysts [21]. 

**Figure 11:**
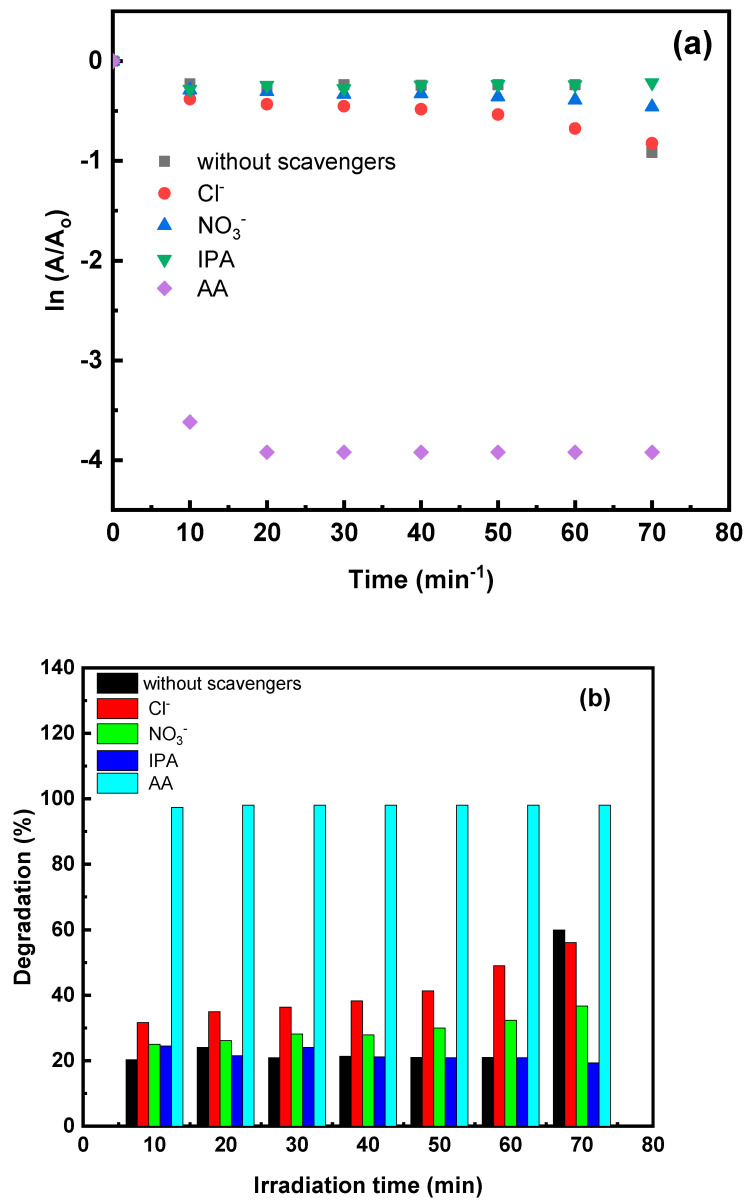
Trapping experiments of active species for the MB photocatalytic reaction using 7.5% Nd_2_O_3_ doped CuO under visible light irradiation using 200 mM scavengers, (**a**) kinetics, (**b**) Degradation efficiency.

The scavenging of active characters responsible for photodegradation in the presence of 7.5% Nd_2_O_3_ doped CuO nanoparticle is examined as demonstrated in Figure 11a. The scavengers applied such as NaCl for h^+^, NaNO_3_ for e^−^, IPA for OH^.^ and Ascorbic acid for $O_{2}^{-.}$. From the data shown in Figure 11b, the use of NaCl, NaNO_3_, IPA, and Ascorbic acid as scavengers is responsible for eliminating MB contaminants of around 56, 36.7, 19.4, and 98 percent. The most efficient agent in eliminating pollutants may be regarded as hydroxy radicals. Sahar Zinatloo et al. [38] reported that in the presence of Nd_2_O_3_-SiO_2_, hydroxyl radicals were the most efficient agent for the degradation of methyl violet. 

#### 3.4.8. Mechanism of Photocatalysis for Nd_2_O_3_ Doped CuO Nanostructured

**Scheme 4:**
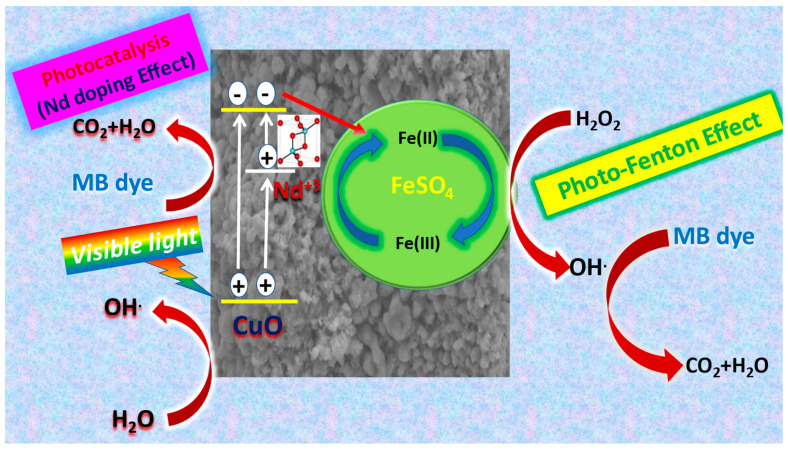
A suggested mechanism for the photocatalysis by (Nd_2_O_3_ doped CuO catalyst)and photo-Fenton by (FeSO_4_) degradation of MB.

Photocatalysis and the photo-Fenton procedure are responsible for MB degradation. During the reaction produced hydroxyl radicals that are represented in Scheme 4. After irradiation, an electron has been excited, and holes are generated to react with H_2_O and dissolved O_2,_ producing ${\mathrm{OH}}^{.}$ radicals that cause MB degradation [39,40]. The proposed degradation process for MB pollutants can be represented as:

(12) $$\mathrm{MB}+h\upsilon \to \mathrm{MB}\;\left(e^{-}+h^{+}\right),$$

(13) $$h^{+}+H_{2}O\to {\mathrm{OH}}^{.}+H^{+},\;$$

(14) $$2h^{+}+2H_{2}O\to H_{2}O_{2}+2H^{+}$$

(15) $$H_{2}O_{2}\to 2{\mathrm{OH}}^{.},$$

(16) $$e^{-}+O_{2\;}\to O_{2}^{-.},$$

(17) $$O_{2}^{-.}+H_{2}O+H^{+}\to H_{2}O_{2}+O_{2,\;}$$

(18) $$H_{2}O_{2}\to 2{\mathrm{OH}}^{.},$$

(19) $${\mathrm{OH}}^{.}+\mathrm{MB}\;\mathrm{contaminant}\to \;\mathrm{Degradation}\;\mathrm{products},\;$$

## 4. Conclusions

The combustion method was applied to synthesize pure CuO, and different concentrations of Nd_2_O_3_ incorporated CuO nanocomposites. The addition of neodymium to CuO improved its structural and morphological characteristics and improved the photocatalytic activities for the apparent photodegradation of MB dye. The crystallinity of the synthesized samples was shown through XRD analysis. The first phase’s average crystallite domain size ranges from 22.081 to 30.109 nm, while the second phase ranges from 27.180 to 36.744 nm. In addition, ε and δ have been calculated. SEM investigation indicated that particles are aggregated homogenously with almost spherical forms. ζ_av_-potentials data-enhanced via rising Nd_2_O_3_ content up to 7.5% Nd_2_O_3_ doped CuO nanoparticle reaching ~27.0 mV with the conductivity of 0.024 mS/cm. The as-prepared 7.5% Nd_2_O_3_ doped CuO nanoparticle exhibits the optimum photocatalytic efficiency comes 90.8% within 80 min with a degradation rate of 0.02274 min^−1^ for MB dye. In comparison, the photocatalytic efficiency was 87% within 80 min with the rate of 0.01683 min^−1^ for MB by CuO.

The degradation efficiency enhances at pH 10 to be 99% with a rate of degradation of 0.082 min^−1^. The stability of the Nd_2_O_3_ doped CuO nanoparticle has been examined through recycling. The results of trapping experiments were indications that the hydroxyl radicals are responsible for the elimination of pollutants. Finally, Nd_2_O_3_-doped CuO nanostructured was a promising material under visible light to treat vast quantities of wastewater.

## Figures and Tables

**Table 1:** The values of diffraction peaks of the as-prepared NPs.

Sample	Line	Position, *2*θ	Intensity (Counts)	Line	Position, *2*θ	Intensity (Counts)
**Pure CuO**	**1**	**35.6**	**901**	**5**	**58.3152**	**149**
**1% Nd_2_O_3_-doped CuO**			**784**			**119**
**2.5% Nd_2_O_3_-doped CuO**			**580**			**88**
**5% Nd_2_O_3_-doped CuO**			**507**			**64**
**7.5% Nd_2_O_3_-doped CuO**			**364**			**51**
**10% Nd_2_O_3_-doped CuO**			**286**			**40**
**Pure CuO**	**2**	**38.7**	**982**	**6**	**61.6**	**245**
**1% Nd_2_O_3_-doped CuO**			**803**			**197**
**2.5% Nd_2_O_3_-doped CuO**			**624**			**148**
**5% Nd_2_O_3_-doped CuO**			**507**			**121**
**7.5% Nd_2_O_3_-doped CuO**			**350**			**76**
**10% Nd_2_O_3_-doped CuO**			**281**			**67**
**Pure CuO**	**3**	**48.8**	**311**	**7**	**66.3**	**211**
**1% Nd_2_O_3_-doped CuO**			**241**			**152**
**2.5% Nd_2_O_3_-doped CuO**			**182**			**116**
**5% Nd_2_O_3_-doped CuO**			**156**			**90**
**7.5% Nd_2_O_3_-doped CuO**			**96**			**41**
**10% Nd_2_O_3_-doped CuO**			**72**			**53**
**Pure CuO**	**4**	**53.5**	**104**	**8**	**68.0**	**203**
**1% Nd_2_O_3_-doped CuO**			**71**			**158**
**2.5% Nd_2_O_3_-doped CuO**			**47**			**109**
**5% Nd_2_O_3_-doped CuO**			**32**			**77**
**7.5% Nd_2_O_3_-doped CuO**			**25**			**54**
**10% Nd_2_O_3_-doped CuO**			**10**			**52**

**Table 2:** The computed mean values of the grain size, dislocation, and strain from the XRD spectra, for all prepared Nd_2_O_3_-CuO nanocomposites.

Samples	Phases	Mean Values of the Grain Size, (nm)	Mean Values of Dislocation Density, (nm)^2^	Mean Values of Lattice Strain
**Pure CuO**	**Phase1, Pure CuO**	29.687	1.262 × 10^−3^	1.213 × 10^−3^

**1% Nd-doped CuO**	**Phase1, Pure CuO**	22.089	2.089 × 10^−3^	1.579 × 10^−3^
	**Phase 2, Nd_2_O_3_**	36.744	1.187 × 10^−3^	1.124 × 10^−3^

**2.5% Nd-doped CuO**	**Phase1, Pure CuO**	30.969	1.582 × 10^−3^	1.305 × 10^−3^
	**Phase 2, Nd_2_O_3_**	27.180	1.636 × 10^−3^	1.356 × 10^−3^

**5% Nd-doped CuO**	**Phase1, Pure CuO**	26.809	1.679 × 10^−3^	1.384 × 10^−3^
	**Phase 2, Nd_2_O_3_**	35.557	9.307 × 10^−3^	1.033 × 10^−3^

**7.5% Nd-doped CuO**	**Phase 1, Pure CuO**	30.109	1.439 × 10^−3^	1.267 × 10^−3^
	**Phase 2, Nd_2_O_3_**	30.154	1.315 × 10^−3^	1.218 × 10^−3^

**10% Nd-doped CuO**	**Phase1, Pure CuO**	27.295	2.141 × 10^−3^	1.522 × 10^−3^
	**Phase 2, Nd_2_O_3_**	36.609	1.518 × 10^−3^	1.208 × 10^−3^


**Table 3:** The average particle size of all prepared samples.

Sample	Particle Size Average Value (nm)
**CuO**	**19**
**1%Nd_2_O_3_**	**22**
**2.5%Nd_2_O_3_**	**39**
**5%Nd_2_O_3_**	**36**
**7.5%Nd_2_O_3_**	**17**
**10%Nd_2_O_3_**	**26**

**Table 4:** The rate constants (min^−1^) for all photodegradation.

Samples	K, (min^−1^)
**Pure CuO**	0.01683
**1% Nd_2_O_3_-CuO**	0.01743
**2.5% Nd_2_O_3_-CuO**	0.02122
**5% Nd_2_O_3_-CuO**	0.01672
**7.5% Nd_2_O_3_-CuO**	0.02274
**10% Nd_2_O_3_-CuO**	0.01935
**7.5% Nd_2_O_3_-CuO, 1 mL H_2_O_2_**	0.00304
**7.5% Nd_2_O_3_-CuO, 2 mL H_2_O_2_**	0.00313
**7.5% Nd_2_O_3_-CuO, 3 mL H_2_O_2_**	0.00312
**7.5% Nd_2_O_3_-CuO, 4 mL H_2_O_2_**	0.00697
**7.5% Nd_2_O_3_-CuO, 5 mL H_2_O_2_**	0.0114
**7.5% Nd_2_O_3_-CuO, 2 mL H_2_O_2_, pH = 2**	0.00161
**7.5% Nd_2_O_3_-CuO, 2 mL H_2_O_2_, pH = 4**	0.00115
**7.5% Nd_2_O_3_-CuO, 2 mL H_2_O_2_, pH = 6**	0.03666
**7.5% Nd_2_O_3_-CuO, 2 mL H_2_O_2_, pH = 8**	0.05734
**7.5% Nd_2_O_3_-CuO, 2 mL H_2_O_2_, pH = 10**	0.08209

**Table 5:** Comparison of photocatalytic degradation of MB and different dyes in the presence of Nd_2_O_3_-doped metal oxide with other previous work samples.

Photocatalysts	Method of Preparation	Organic Solution	Irradiation Time	Lamp Source	% Degradation	k, (min^−1^)	Refs.
**7.5% Nd-doped** **CuO**	Combustion	MB	80 min	Visible light	90.8%	0.0227	**Present work**
**7.5% Nd-doped** **CuO at pH 4**	Combustion	MB	80 min	Visible light	99%	0.082	**Present work**
**Nd_2_O_3_-doped ZnO**	Hydrothermal method	MB	60 min	UV-light	96%	0.3145	**[30]**
**Ag/Nd_2_O_3_-ZnO**	Hydrothermal method	MB	30 min	Visible-light	98.12%	-------	**[17]**
**Nd_2_O_3_-doped SnO_2_**	Sol gel method	MB	4 h	Visible-light	93.1%	0.615 h^−1^	**[31]**
**Nd_2_O_3_-doped ZnO**	Co-precipitation	indigo carmine	210 min	Visible-light	74%	3.87 × 10^−3^	**[32]**
**Nd_2_O_3_-ZnO-GO** **(0.3% Nd)**	Co-precipitation	indigo carmine	210 min	Visible-light	95%	1.36 × 10^−2^	**[32]**
**Nd–TiO_2_–GO** **(0.6% Nd)**	Sol gel method	indigo carmine	180 min	Visible-light	92%	13.42 × 10^−3^	**[33]**
**Nd-doped ZnO**	Sol-gel method	MB	25 min	UV-light	98%	-------	**[34]**
**3 mol%Ce^3+^-doped CuO**	Sonochemical method	MB	180 min	Visible-light	98%	-------	**[35]**
**Nd-TiO_2_-C**	Sol-gel and impregnation method	MB	160 min	UV-light	100%	--------	**[36]**
**Nd-doped ZnO**	sol-gel method	TC	120 min	Visible-light	100%	--------	**[37]**
**CuO**	Microwave	VB(10^−5^ M) DR(10^−5^ M)	-------	UV-visible irradiation and US radiations	100%	0.09861 0.10587	**[26]**

## Data Availability

The data supporting this study’s findings are available from the corresponding author upon reasonable request.
